# Fungal diversity and drug susceptibility of the oral mycobiome of domestic dogs

**DOI:** 10.3389/fvets.2023.1281712

**Published:** 2023-11-15

**Authors:** Brooke D. Esquivel, Elisa M. Rouse Salcido, Allison M. Schweiker, Brandon L. Holder, Butch KuKanich, Kate S. KuKanich, Theodore C. White

**Affiliations:** ^1^Division of Biological and Biomedical Systems, School of Science and Engineering, University of Missouri – Kansas City, Kansas City, MO, United States; ^2^Department of Anatomy and Physiology, Kansas State University College of Veterinary Medicine, Manhattan, KS, United States; ^3^Department of Clinical Sciences, Kansas State University College of Veterinary Medicine, Manhattan, KS, United States

**Keywords:** oral mycobiome, *Malassezia pachydermatis*, antifungal drug susceptibility, fungal pathogens, commensal fungi, canine mycobiome

## Abstract

The purpose of this study was to characterize the variety and diversity of the oral mycobiome of domestic dogs and to identify the commensal and potentially pathogenic fungi present. Two hundred fifty-one buccal swabs from domestic dogs were obtained and struck onto a chromogenic fungal growth medium that distinguishes between fungal species based on colony color and morphology. After isolating and harvesting single colonies, genomic DNA was extracted from pure cultures. PCR was used to amplify a fungal-specific variable rDNA region of the genome, which was then sent for sequencing. Sequencing results were input into the NCBI BLAST database to identify individual components of the oral mycobiome of tested dogs. Of the 251 dogs swabbed, 73 had cultivable fungi present and 10 dogs had multiple fungal species isolated. Although the dogs did not show signs of oral infections at the time, we did find fungal species that cause pathogenicity in animals and humans. Among fungal isolates, *Malassezia pachydermatis* and species from the genus *Candida* were predominant. Following fungal isolate identification, antifungal drug susceptibility tests were performed on each isolate toward the medically important antifungal drugs including fluconazole, ketoconazole, and terbinafine. Drug susceptibility test results indicated that a large number of isolates had high MIC values for all three drugs. Exploring the oral mycobiome of dogs, as well as the corresponding drug susceptibility profiles, can have important implications for canine dental hygiene, health, and medical treatment. Identifying the microorganisms within the canine mouth can illustrate a common pathway for fungal pathogens of One Health concern to spread from our canine companions to humans.

## Introduction

Pathogenic and opportunistic fungi can cause superficial and mucosal infections, as well as life-threatening systemic diseases in humans and other animals (1–3). However, many fungal species are now recognized as being commensal inhabitants of diverse and complex microbial communities that live on nearly every surface of healthy humans and animals (4). The importance, and relationship to health and disease, of commensal fungal colonization within specific biocompartments like the mouth and gut is an active field of study. A key component to understanding the role of these fungi is to first characterize and define the fungal species composition and biodiversity within individuals and populations. Characterization of the mycobiome of domestic animals by defining fungal colonizers could be an important dataset for not only animal health but also that of their human caregivers. Colonization by opportunistic fungal pathogens has the potential to lead to clinically relevant disease. We focused our study on characterizing the oral mycobiome of a large population of healthy domestic dogs.

Fungi are diverse, ubiquitous, and can survive in a variety of environments including on food, in soil, in the air, as well as on plant and animal hosts (5, 6). Because dogs act as a segue between indoor and outdoor environments, as they explore the world with their mouths, they may expose human households to environmental fungi that would not otherwise be encountered. Therefore, in addition to identifying oral colonizing fungi, detecting transient occupiers would also be pertinent for zoonotic disease transmission potential. Regarding the ever-increasing numbers of immune compromised humans, monitoring the fungal inhabitants of dogs’ mouths might illuminate a route of human exposure to fungal pathogens of One Health concern, and even be a predictor of environmentally emerging human infections.

Much work has been done to characterize the oral mycobiome of humans using a variety of techniques and studying an assortment of participant populations (7). One such analysis using massively parallel, high throughput metagenomics techniques revealed heterogeneity of fungi present in individual human mouths, as well as high interindividual fungal variability (8). The researchers identified *Malassezia*, *Epicoccum* and *Candida* as the most abundant fungal genera found in healthy adult mouths (8). Species of the *Malassezia* genus are well-known cutaneous colonizers of humans and animals, often causing opportunistic infections of the skin (9). However, prior to this large-scale oral mycobiome study, *Malassezia* had not been recognized as a component of healthy mucosal flora (7, 8).

Limited studies have been done to lay the foundation for characterizing the canine oral mycobiome. One group of researchers have used a metagenomics approach with direct DNA extraction from saliva and found *Cladosporium* and *Malassezia* species to be the most frequently encountered (10). Metagenomics approaches can provide great sensitivity to ensure low-abundance and non-culturable fungi are detected. However, this genomics-only approach does not allow for further analysis of the fungal isolate in terms of morphology, drug susceptibility and cell-biology. An important part of the oral mycobiome characterization includes drug susceptibility analysis which can only be done using cultured fungi. Therefore, we have restricted our analysis to culturable fungi. With our analysis, correlations can be made between the composition of the mycobiome and drug susceptibility or resistance, which could help predict potential treatment challenges in these dogs if clinical disease or serious infection develops.

Another group analyzing the canine oral mycobiome of fifty street dogs used a commercial kit based on carbohydrate assimilation pattern (auxanogramme) to identify yeast species present (11). These kits are designed to test for the presence of the most commonly encountered yeast species and may not be useful for detecting less common fungal groups. These researchers found the majority of isolates to be from the *Candida* genus, followed by Trichosporon spp. and 1 isolate of *Malassezia pachydermatis*. Further examination of the drug susceptibility of those fungal isolates determined that many of the canine fungal isolates had elevated MICs to fluconazole and amphotericin B, while presenting sensitivity to voriconazole (11).

Our study aimed to further elucidate the oral mycobiome in a large population of healthy domestic dogs, using a combination of culture-based mycological diagnostics techniques with speciation by ribosomal DNA (rDNA) genomic analysis, while maintaining the intact organism for further analysis. We determined drug susceptibility profiles of each fungal isolate to the common antifungals approved for human and canine therapy, including fluconazole, ketoconazole and terbinafine (12–14). We discovered a diversity of fungi present in the mouths of dogs including fungi commonly found in the soil and environment, many with reduced drug sensitivity. More importantly, we found fungi that can cause disease in humans, including some listed in the World Health Organization fungal priority pathogens list (15). This research, combined with others, works to build a healthy state baseline for the future study of fungal communities forming the oral mycobiome of dogs.

## Materials and methods

With University of Missouri – Kansas City (UMKC) IACUC approval and concurrence by Kansas State University (KSU) IACUC, buccal swabs were obtained from the mouths of 209 regional shelter dogs and 42 purpose-bred beagles from February 2021 to September 2022. Veterinarians (KSK or BSK) visited the shelter weekly and took samples from new dog arrivals at this regular interval. Detailed dog information such as past medical history, previous cohabitation with other animals, and former diet is not available prior to the dog entering the shelter. At the shelter, dogs are provided a balanced and complete maintenance dog food and are housed in single runs. Assuming behavior compatibility, dogs have daily walks and play time outside when they may interact with other dogs at the shelter. Available medical records were reviewed by a single investigator (KSK). No dog was known to be receiving antifungal therapy at the time of sampling.

A single buccal specimen was collected from each dog by rolling an Eswab culturette (Thermo Scientific™ R723480) between their cheek and teeth; all specimens were collected by one of two members of the research team (BK or KSK). The commercially available swabs include modified Liquid Amies media designed for easy containment and transport of the swab to the laboratory. All swabs were shipped overnight to the microbiology lab (TCW) where the specimens were struck onto CHROMagar *Candida* (16). This selective and differential media contains chloramphenicol to inhibit growth of oral bacteria as well as chromogenic components that allow for the distinction between various fungal species by color and colony morphology. After incubation at 30°C for 3–7 days, colonies with unique color or morphology were restruck on CHROMagar *Candida* three sequential times to ensure a single species was isolated.

Each isolate was cultured in Yeast Extract Peptone Dextrose [(YEPD) 10 g yeast extract, 20 g peptone, and 20 g dextrose per liter] rich media at 30°C shaking for 48 h at which time glycerol stocks were created by adding glycerol at a 30% final concentration. Each isolate was frozen at −80°C for long-term storage, creating a library of canine oral fungal isolates.

DNA was prepared from the isolates by ethanol precipitation (17), and the rDNA Internal Transcribed Spacer (ITS) of each isolate was amplified by PCR using the primer pair ITS-1 (5’-TCCGTAGGTGAA CCTGCGG-3′) and ITS-4 (5’-TCCTCCGCTTATTGATATGC-3′) which includes the genes ITS-1, the 5.8S gene, and the ITS-2 region (18).

Each PCR product was checked for correct amplicon size (~450 bp) on a 0.8% Tris-acetate-EDTA gel. The remaining product was purified and then sequenced at the University of Missouri - Columbia Genomics Technology Core (Columbia, MO). The resulting sequences were analyzed using NCBI Basic Local Alignment Search Tool (BLAST) (19) to identify each isolate to the species level.

Guidelines for drug susceptibility testing and interpretation for the *Malassezia* genus are not well defined, compared to other yeasts such as those of the *Candida* genus. Traditional broth microdilution assays as described by the Clinical and Laboratory Standard Institute (CLSI) reference assay [20] do not work well with *Malassezia.* Standard RPMI (Roswell Park Memorial Institute) broth is lipid-free, while *M. pachydermatis* needs lipid supplementation for robust growth (21). Instead, we performed drug testing using Modified Dixon’s medium which contains the lipid sources tween, glycerol, and ox bile [36 g Malt extract, 10 g Mycological peptone, 10 g Desiccated ox bile, 10 mL Tween 60, 4 mL 50% Glycerol per liter, (22)]. The sediments in this media obscure broth microdilution spectroscopic readings, and *Malassezia* tends to form clumps in liquid media, thus the decision was made to determine MIC values based on the E-test method and spot test assays using solid media. Additionally, some *M. pachydermatis* isolates have a slower growth rate compared to that of *Candida* species, therefore the E-test values for the slower-growing isolates were measured at 48–72 h.

Minimum Inhibitory Concentrations (MIC) for ketoconazole (KTZ) and fluconazole (FCZ) were measured using E-test strips (bioMerieux, Durham, NC, United States) (23). The isolated fungal samples were plated so as to form a lawn of colonies on agar, followed by placement of the KTZ or FCZ Etest. Plates were incubated at 30°C for 24–72 h. The MIC was determined based on the zone of clearing in colony growth around the drug test strip. Etest strips are not available for terbinafine (TER). The drug susceptibility of each isolate to TER was measured according to spot test assays. Standardized inoculum were spotted onto the agar plate in 4, 10-fold gradient dots starting with OD_600_ 1.0 and ending with 0.001. The agar contained either no drug, 2 μg/mL or 4 μg/mL TER. Fungal dot growth at each drug concentration was compared with the dot growth of that isolate on the no-drug control plate. For all fungal isolates, designations of drug-Resistant, Intermediate, or Susceptible were extrapolated from breakpoints (Table 1b) assigned to the well-studied human isolates of *C. albicans*, because canine CLSI breakpoints are not available for these fungal species and antifungal medications (20, 24, 25).

Taxonomy hierarchy tree (Figure 3) was created using Microsoft Power Point. Venn diagrams comparing drug-resistant or drug-susceptible isolates (Figure 4) were created using Venny 2.1.0 (26).

## Results

**Figure 1:**
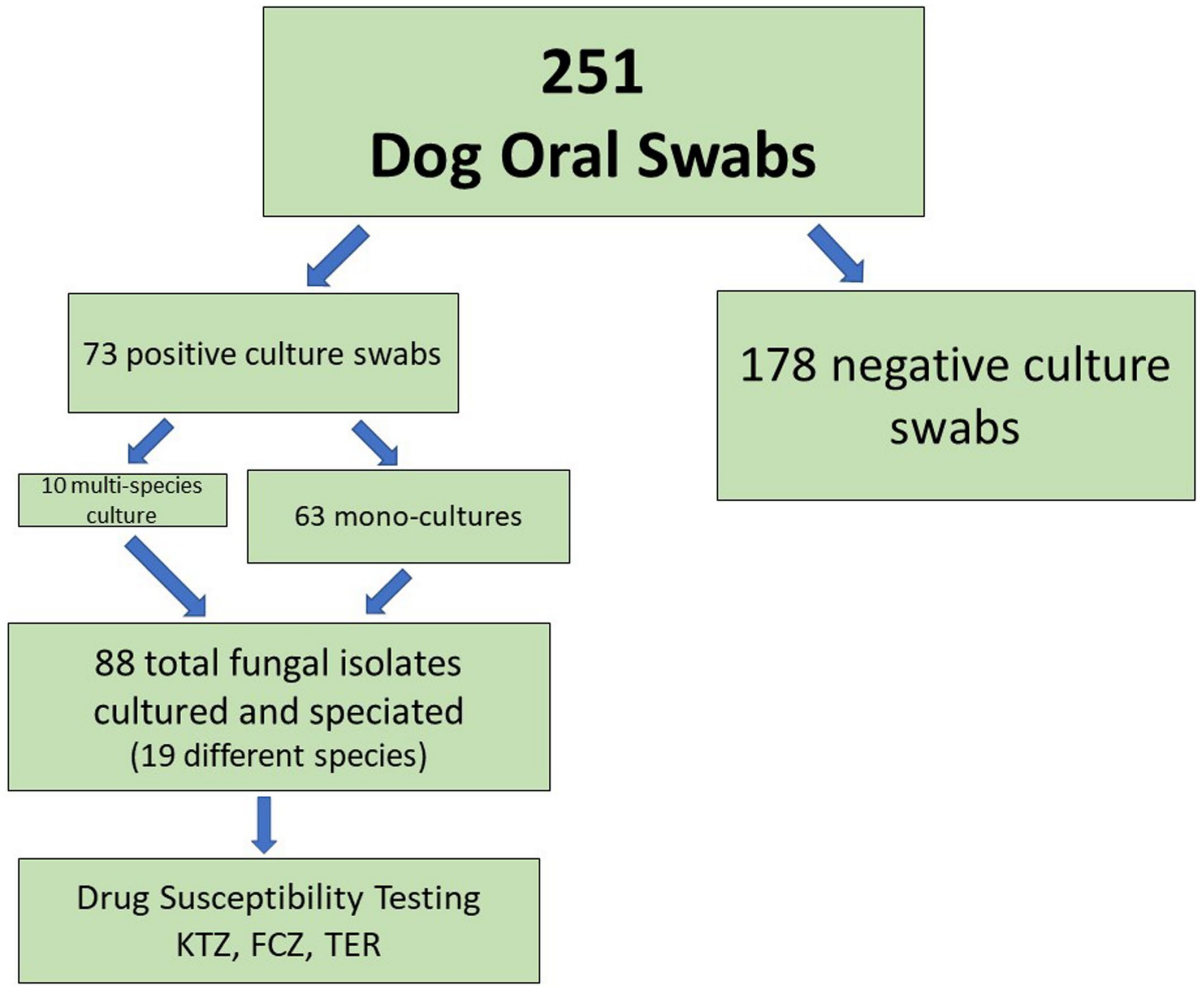
Oral Fungal Isolate Procurement Overview.

**Figure 2:**
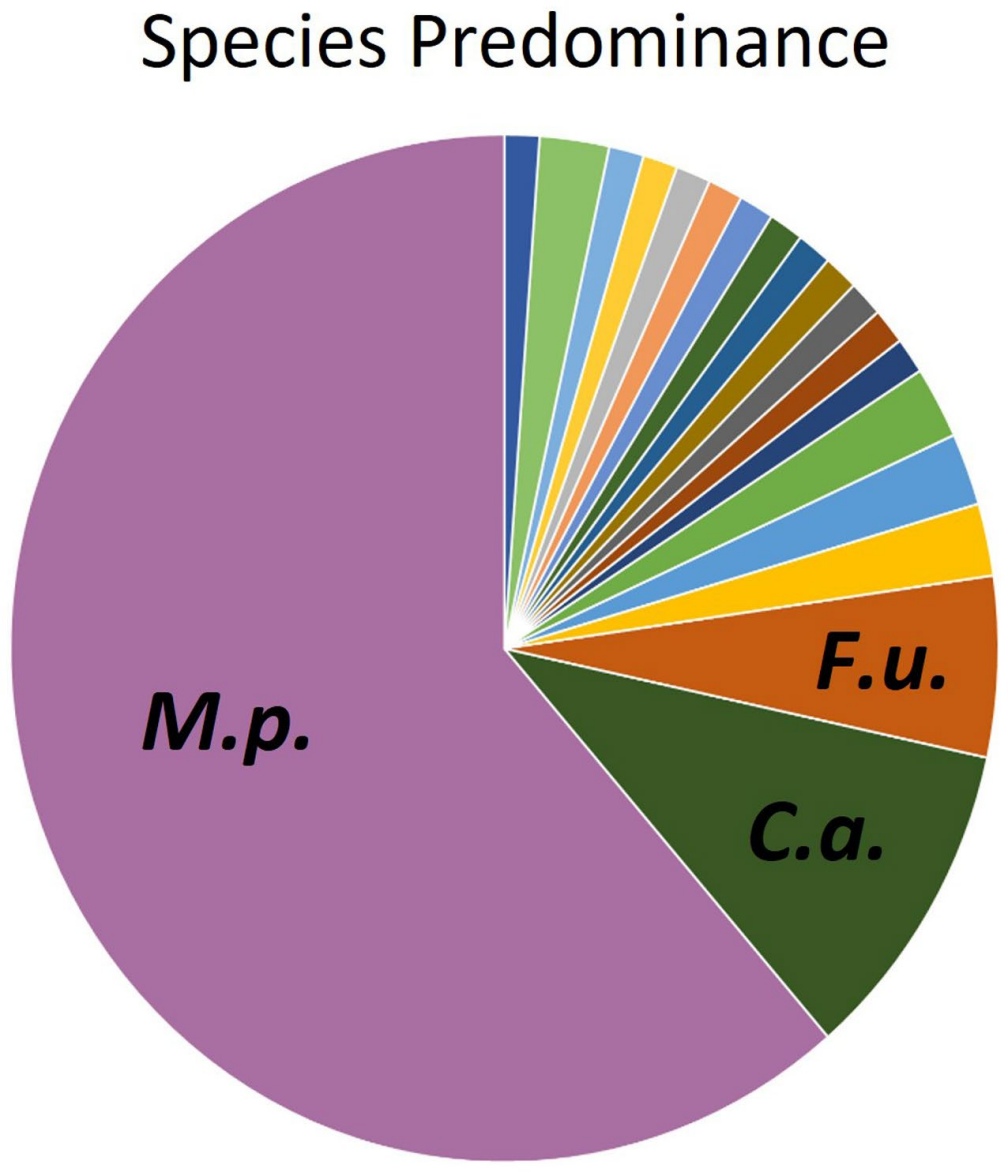
Distribution of fungal species isolated from oral swabs of domestic dogs. M.p., *Malassezia pachydermatis* (61.3%); C.a., *Candida albicans* (10.2%), F.u., *Filobasidium uniguttulatum* (5.7%).

**Figure 3:**
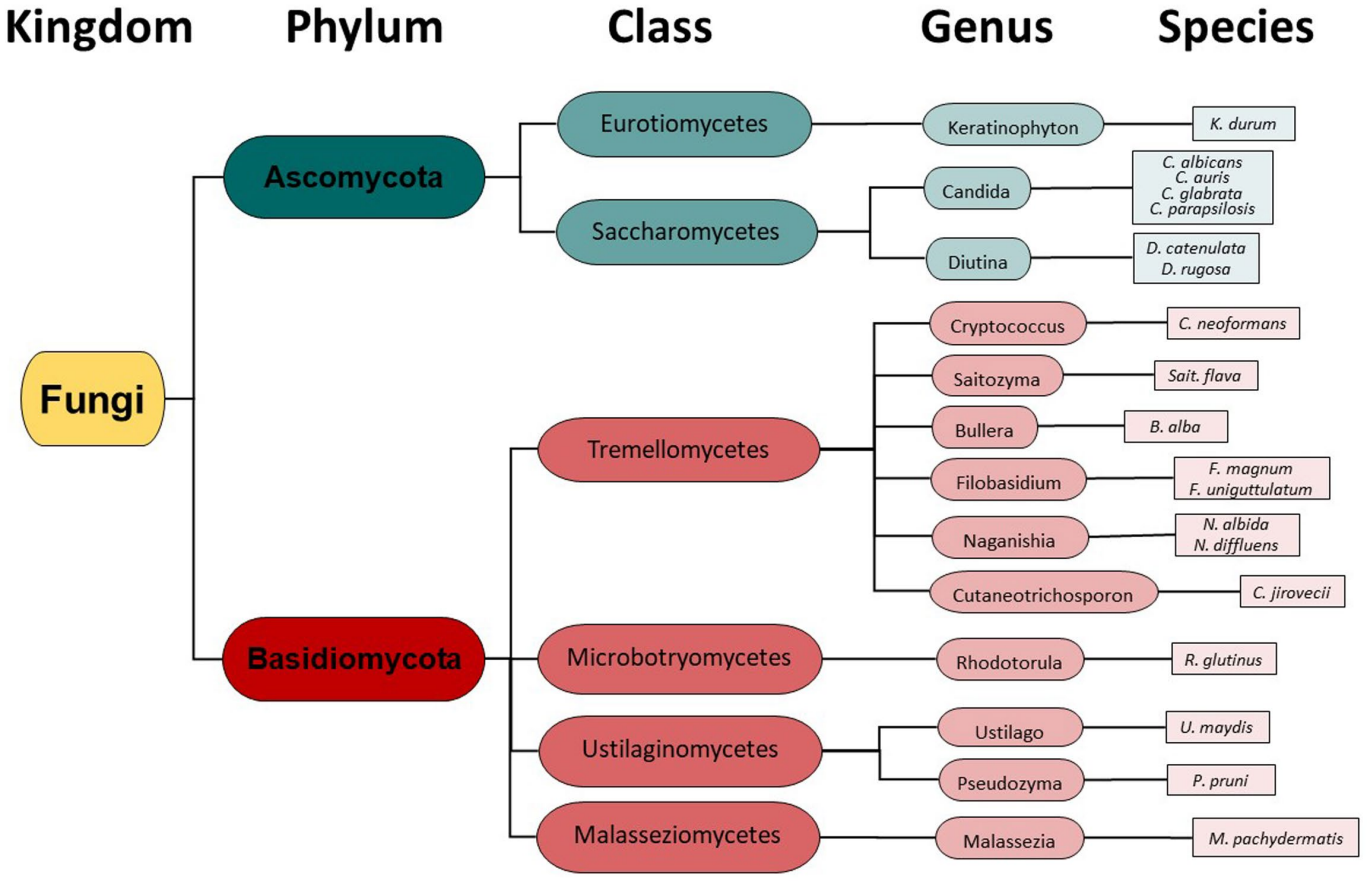
Fungal Taxonomy groupings found in the canine oral cavity mycobiome.

Of 251 dogs analyzed, we were able to cultivate fungal species from the oral swabs of 73 dogs (29%, Figure 1, Table 2). The majority of swabs produced no fungal colonies when struck on CHROMagar *Candida*. Sixty-three dog swabs produced a homogeneous culture with a single unique fungal isolate, while ten dogs were found to have more than one fungal species co-colonizing the oral cavity. One dog was found to have four separate fungal species (Supplemental Sheet 1 and submitted manuscript). In total, we were able to isolate, culture and identify at the species level 88 unique fungal isolates from the oral swabs (Figures 1**–**3, Table 2). Colony color and morphology of each isolate were recorded and can be found in Supplemental Sheet 1.

Instead of conventional mycological diagnostic methods, we relied on amplification and sequencing of the ribosomal DNA (rDNA) that includes the genes ITS-1, the 5.8S gene, and the ITS-2 region (18). ITS-1 and 2 are rapidly evolving regions that harbor enough variability to allow taxonomic discrimination between most fungal species (27). Sequencing results revealed a diversity of fungi coming from two different phyla- Ascomycota and Basidiomycota (Figure 3), 6 different classes, 13 different genera, and finally a total of 19 different fungal species. From Figure 3, it is clear that the majority of different species were from the Basidiomycota, rather than the Ascomycota. The majority of canine oral fungal isolates were the species *Malassezia pachydermatis* (Table 2, Figure 2). The next most commonly isolated species were *Candida albicans* and *Filobasidium uniguttulatum*, which is related to *Cryptococcus.* There were a variety of other fungal species isolated in only two or even single dogs.

Drug susceptibility testing was performed on Modified Dixon’s agar to support robust growth of the large percentage of *M. pachydermatis* isolates. Susceptibility to the azole antifungals fluconazole (FCZ) and ketoconazole (KTZ) were tested using Etests (Table 1a) as described in Material and Methods. FCZ and KTZ are important human and animal antifungal drugs (28). Terbinafine (TER) is a common allylamine antifungal used in veterinary medicine, often used for treatment of *Malassezia* infections (28); however since TER Etests are not available, dot drug susceptibility assays were performed for this antifungal (Table 1a) as described in Material and Methods. The qualification of an isolate as Resistant, Intermediate, or Susceptible to a drug was determined using the cutoff values described in Table 1b (24, 25). One isolate of *Keratinophtyon durum*, was unable to be revived from the frozen glycerol stock and so only 87 isolates were tested for drug susceptibility. Drug susceptibility phenotypes for each specific isolate can be found in Supplemental Sheet 2.

**Table 1a:** Percent of fungal isolates (all species) from oral mycobiome of domestic dogs susceptible, intermediate, and resistant to fluconazole (FCZ), ketoconazole (KTZ), and terbinafine (TER) based on cutoff values (breakpoints) established for human *Candida albicans* isolates (17, 18).

	FCZ	KTZ	TER
Susceptible	36.8%	8.0%	43.7%
Intermediate	24.1%	59.8%	10.3%
Resistant	39.1%	32.2%	46.0%

**Table 1b:** Drug susceptibility cutoff values (24, 25).

	FCZ	KTZ	TER
Susceptible	≤ 8 μg/mL	≤ 0.125 μg/mL	≤ 2 μg/mL
Resistant	≥ 64 μg/mL	≥ 1 μg/mL	≥ 4 μg/mL

There was a range of drug susceptibilities to all drugs tested between the isolates (Tables 1a,b, 2). Of the 54 *M. pachydermatis* isolates, 25.9% were resistant to FCZ, 11.1% resistant to KTZ, and 13.0% resistant to TER (Table 2). The majority of *C. albicans* isolates showed resistance to all drugs tested. Of the nine *C. albicans* isolates, 77.8% were resistant to FCZ, 88.9% were resistant to KTZ, and 100% were resistant to TER. All isolates of *F. uniguttulatum* were resistant to the drugs tested. The overall percent of resistant isolates for FCZ, KTZ, and TER were 39.1, 32.2 and 46.0%. For FCZ and TER, the number of isolates were evenly split between susceptible and resistant MIC profile (Tables 1a,b, 2). For KTZ, the majority of isolates had elevated MICs that fell within the intermediate range of susceptibility (59.8%), while the minority fell below the susceptibility cutoff value (8.0%).

**Table 2:** Fungal isolate species identification and drug resistance from the oral cavity of domestic dogs (*N* = 73); 10 dogs had more than one fungal species co-colonizing the oral mycobiome.

Species	# Isolates	% Isolates	# (%) FCZ Resistant	# (%) KTZ Resistant	# (%) TER Resistant
*Malassezia pachydermatis*	54	61.3	14 (25.9)	6 (11.1)	7 (13.0)
*Candida albicans*	9	10.2	7 (77.8)	8 (88.9)	9 (100)
*Filobasidium uniguttulatum*	5	5.7	5 (100)	5 (100)	5 (100)
*Bullera alba*	2	2.3	0	1 (50)	2 (100)
*Candida parapsilosis*	2	2.3	0	0	2 (100)
*Diutina rugosa*	2	2.3	0	0	2 (100)
*Ustilago maydis*	2	2.3	0	2 (100)	2 (100)
*Candida auris*	1	13.6	1 (100)	1 (100)	1 (100)
*Candida glabrata*	1		0	0	1 (100)
*Saitozyma flava*	1		1 (100)	1 (100)	1 (100)
*Cryptococcus neoformans*	1		1 (100)	0	1 (100)
*Diutina catenulata*	1		0	0	1 (100)
*Filobasidium magnum*	1		1 (100)	1 (100)	1 (100)
*Keratinophyton durum*	1		N/A	N/A	N/A
*Naganishia albida*	1		1 (100)	1 (100)	1 (100)
*Naganishia diffluens*	1		1 (100)	1 (100)	1 (100)
*Pseudozyma pruni*	1		1 (100)	1 (100)	1 (100)
*Rhodotorula glutinis*	1		1 (100)	0	1 (100)
*Cutaneotrichosporon jirovecii*	1		0	0	1 (100)
Total Isolates	88*	100	34 (39.1)	28 (32.2)	40 (46)

*1 isolate was not tested so % calculations based off of 87 total isolates tested.

**Figure 4:**
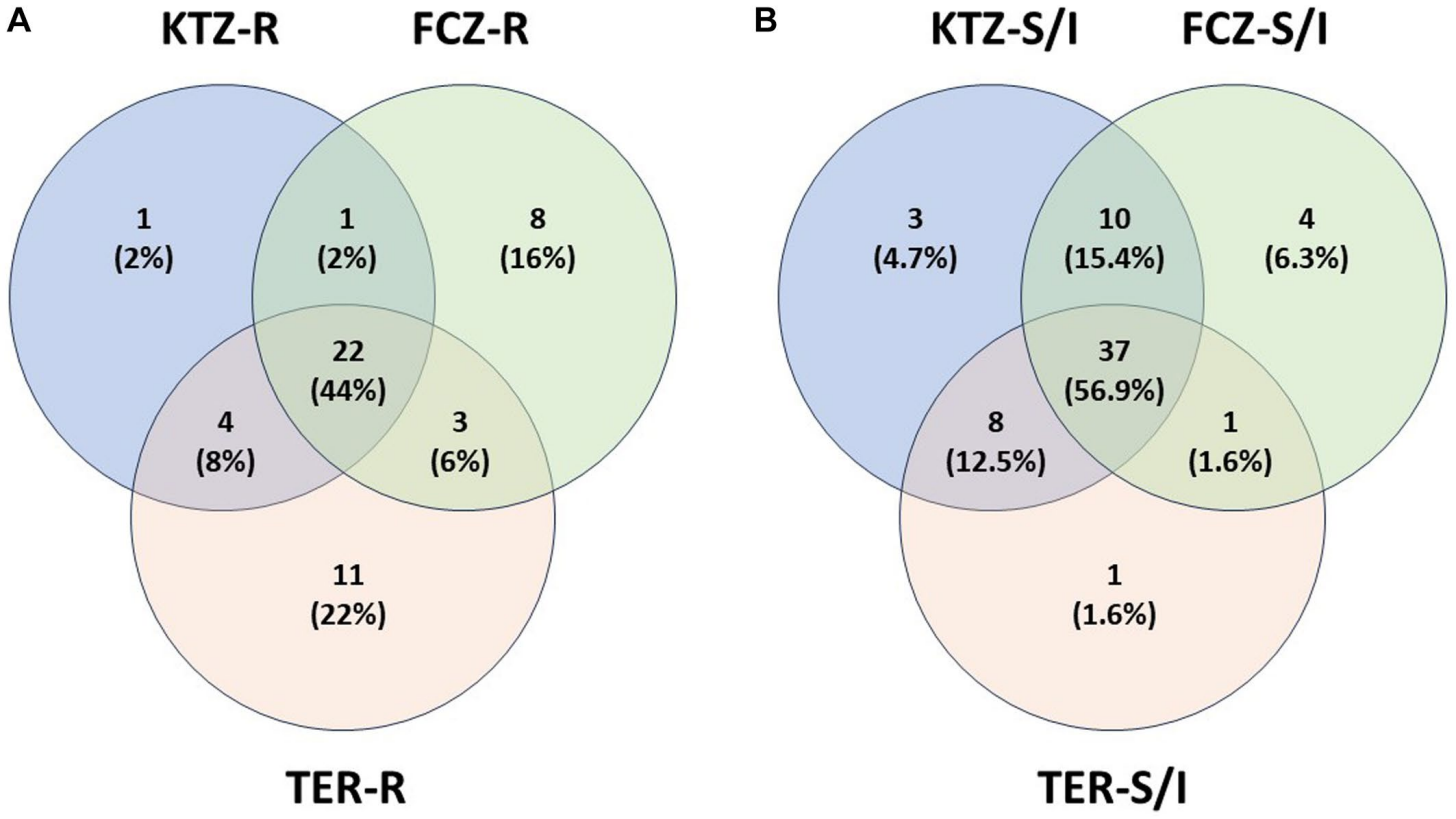
Isolate Drug Resistance and Susceptibility Overlap. **(A)** Isolate multi-drug resistance. Isolates that are resistant (R) to at least one drug were plotted with other resistant isolates, showing percent of multidrug resistance isolates. **(B)** Isolate overlap in Susceptible or Intermediate phenotypes. Isolates that are susceptible or intermediate (S/I) to at least one drug were plotted with other susceptible or intermediate isolates, showing overlap of isolate susceptibility between the drugs.

Isolates that were resistant to at least one drug were analyzed for cross-resistance to the other two drugs and plotted in a Venn Diagram (Figure 4A). Twenty-two isolates (44%) were resistant to all three antifungals. Nearly all isolates that were resistant to KTZ (28) were also resistant to other drugs (27 of 28). There were some isolates that were resistant to TER or FCZ alone, while still susceptible to the other two drugs (Figure 4A). In total, 60% of isolates were multi-drug resistant (Figure 4A). A similar diagram was created with the isolates that had a Susceptible (Sus) or Intermediate (Int) phenotype for any of the three drugs (Figure 4B) the majority of isolates that are susceptible to one drug are susceptible to all 3 drugs (56.9%).

## Discussion

We identified a diversity of fungi present in the mouths of dogs including agricultural fungi and animal pathogens, comprising 19 unique fungal species (Table 2, Figure 3). Importantly, we also found fungi that can potentially cause disease in humans and dogs. In fact, some of the identified fungi are listed on the WHO Priority Pathogens Report (15) such as *Candida auris*, *Candida albicans*, *Candida parapsilosis* and *Cryptococcus neoformans* (Table 2, Figure 3).

The majority of canine buccal swabs were culture negative (Figure 1). This was not especially surprising since we know fungi make up a small percentage of human and animal microbiomes (29). This analysis was performed using culture-based isolation by streaking swabs onto solid media, rather than a metagenomics approach with DNA extracted from saliva. It is possible that many mycobiome organisms are not culturable outside of their specialized ecological niches, or that our choice of culture medium (CHROMagar *Candida*) did not support growth of many fungal species with additional nutrient requirements. So, our culture-based isolation most likely missed some low-abundance or un-culturable fungi, but the culture of the fungal isolate was necessary for drug susceptibility testing and other potential cell-biology analyses for which we need the living organism.

*Malassezia pachydermatis* was the most abundant species isolated from dog mouths (Table 2, Figure 2). *Malassezia* spp. are opportunistic pathogens and under favorable conditions, they can cause fully symptomatic infection (9). *Malassezia pachydermatis* is a known pathogen of the ears and skin in dogs (30). Other species from the *Malassezia* genus can cause skin conditions in humans (31, 32). Our findings agree with previous mycobiome surveys in humans and dogs that also found *Malassezia* to be a commensal on mucosal surfaces of healthy animals (8, 33). It would be interesting to determine if the oral, cutaneous and ear canal *M. pachydermatis* isolates of one animal are all derived from the same strain or have different origins. Related commensal strains in the different locations would seem likely considering dogs lick and groom routinely, but this has not been investigated to our knowledge.

This work also confirms the distinction in the commensal *Malassezia* populations between humans and in the canine oral cavity mycobiome, with *M. pachydermatis* being the most commonly detected species in dogs and *M. restricta* and *M. globosa* being the most common *Malassezia* human commensals (31, 33, 34). *Malassezia pachydermatis* and *M. furfur* are able to grow on CHROMagar *Candida* with no additional lipid supplementation, but we did not detect *M. furfur* from any dogs in our study. It is commonly known that *Malassezia* spp. are lipid-dependent, but *M. pachydermatis* exceptionally is capable of growing on standard mycological media such as Sabouraud agar (SGA) and YEPD agar because these undefined media include peptone (35, 36). Peptone contains a minimum amount of lipids, which allow the adequate growth of the most strains of *M. pachydermatis* (35). However, there could be other, more lipid dependent *Malassezia* species present in the canine oral mycobiome that were not able to be harvested using our culture conditions (35).

The four well-known human pathogens belonging to the *Candida* genus that were isolated are *Candida albicans*, *Candida auris*, *Candida glabrata*, and *Candida parapsilosis*. Species from the *Candida* genus can cause superficial and systemic infections in humans and are the source of cutaneous and urinary tract infections in dogs and cats (37, 38). *Candida auris* is a more-recently emerging human fungal pathogen that often colonizes patients while remaining asymptomatic (39). However, infections from *C. auris* have a high mortality rate because the fungus is often resistant to antifungal treatment (39). The *C. auris* isolate was confirmed by the Centers for Disease Control and Prevention Fungal Disease Laboratory to be *C. auris* and was identified as a novel isolate that is part of the South American Clade (Clade 4; submitted manuscript). This isolate is the first report of *C. auris* found in non-human animals in the United States but is consistent with recent findings of *C. auris* on the skin and ears of stray dogs in India (40). This further emphasizes the role of domestic animals as potential reservoirs of disease for humans and might be an important consideration for safety precautions (e.g., washing hands, discouraging licking – especially of the face, cleaning and disinfecting food and water bowls regularly). Further surveys could help define the relative risk of dogs as a potential source for pathogenic organisms relative to other sources of risk (e.g., foods, environment, wildlife, other humans).

*Cryptococcus neoformans* is a human and animal pathogen that can cause severe central nervous system, respiratory, ocular, and systemic disease in dogs and cats (41). The route of exposure in these cases is inhalation, although other routes have been postulated. As colonization of the respiratory tract with *C. neoformans* is recognized in healthy dogs and cats (42, 43), it is not unexpected to also find it in the mouth, although the clinical significance of this finding, and risk of future disease, is unknown.

Seven species were identified that occasionally cause pathogenicity in humans and are closely related to other human pathogens [*Saitozyma flava (Cryptococcus), Diutina (Candida) rugosa*, *Diutina (Candida) catenulate, Filobasidium (Cryptococcus) magnum*, *Filobasidium (Cryptococcus) uniguttulatum*, *Naganishia (Cryptococcus) albida*, and *Naganishia (Cryptococcus) diffluens*; genus names in parentheses are the former genus name].

Finally, six species that are mostly environmental or agricultural but can rarely cause human infection include *Bullera alba*, *Keratinophyton durum, Pseudozyma pruni, Rhodotorula glutinis, Cutaneotrichosporon jirovecii* and *Ustilago maydis.* The species *Bullera alba* is known to produce an anti-microbial (mycocin) with antifungal activity (44), and it is not surprising that the swab that contained *Bullera alba* did not contain any other fungal species.

Drug susceptibility test results indicated that a large number of isolates had high MIC values for all three drugs KTZ, FCZ and TER, with many exceeding the human *Candida* cutoff value to be considered resistant (Tables 1a,b). Since validated MIC breakpoints are limited to a small number of species and indications, we chose to use *Candida* as a representative for all fungal isolates; however, this extrapolation is a limitation that might not represent the ideal clinical breakpoints for each fungal isolate (*Malassezia, Cryptococcus*, etc.), host species (dogs), and infection location (soft tissue including oral cavity, CNS, etc.). Additionally, results of the drug susceptibility tests do not necessarily translate into clinical treatment outcome (13) and the isolates were not causing an active infection in any of the dogs. But it is still important to note the elevated MIC values and evidence for multi-drug resistance in these commensals (Figure 4A). These data can also help assess the risk and predicted efficacy of using antifungal drugs in dogs for effective treatment, including prophylaxis and long-term treatments (45–49).

KTZ and FCZ target the fungal enzyme Erg 11 while TER targets Erg 1 (12, 50). All three antifungals target the fungal ergosterol biosynthesis pathway, so mechanisms that provide resistance to one drug may provide resistance to the others. For example, gain of function mutations in transcription factors that regulate genes involved in ergosterol biosynthesis, or overexpression of efflux transporter genes may lead to reduced susceptibility to these antifungals (51, 52). The levels of resistance can simply be due to intrinsic resistance of the strains or species. Alternatively, acquired resistance in these isolates could have developed from exposure to environmental antifungals before the isolates colonized the dogs, or dog to dog transfer – the dogs could have been exposed to the resistant isolates from other dogs during their shelter stay. The resistance mechanisms could also have been transmitted to the dogs from humans, such as previous owners, animal control or shelter personnel who either harbor resistant fungi or have received antifungal therapy. Additionally, medical records from shelter dogs are inherently incomplete, and some dogs might have received antifungal therapy in the past that was unknown to the investigators. Regardless, this illustrates a mechanism by which fungal pathogens that are resistant to antifungals in a non-human host can increase the potential for treatment failure in human infections even though the human has had no previous drug exposures. Expanded studies could be done to determine if a true correlation exists between cohabitation and sharing of mycobiota between human and animal members of the same household.

Oral mycobiome composition is most likely influenced by a number of factors that could be determined with further studies to correlate the fungal species present and canine age, health, breed, diet, geographic region, and even shelter vs. private residence. For example, expansion of the research to include regional comparisons of dog oral mycobiomes might elucidate changing areas of endemicity of environmental fungal pathogens and correlations to changing climate conditions. The microbial community present in the oral mucosa may forecast emerging human and animal fungal pathogens. This work can be used as a model for future exploration of the mycobiome of pets, livestock, and other animals.

## Data availability statement

The original contributions presented in the study are included in the article/Supplementary material, further inquiries can be directed to the corresponding author.

## Ethics statement

The animal studies were approved by University of Missouri Kansas City and Kansas State University Institutional Animal Care and Use Committees. The studies were conducted in accordance with the local legislation and institutional requirements. Written informed consent was obtained from the owners for the participation of their animals in this study.

## Author contributions

BE: Conceptualization, Data curation, Funding acquisition, Investigation, Methodology, Project administration, Supervision, Writing – original draft, Writing – review & editing. ER: Data curation, Investigation, Formal analysis, Writing – review & editing. AS: Data curation, Investigation, Writing – review & editing. BH: Investigation, Writing – review & editing. BK: Supervision, Writing – review & editing, Conceptualization, Funding acquisition, Project administration, Resources. KK: Methodology, Validation, Writing – original draft, Conceptualization, Supervision, Writing – review & editing. TW: Formal analysis, Funding acquisition, Resources, Conceptualization, Methodology, Supervision, Validation, Writing – original draft, Writing – review & editing.
